# Rare Disease Diagnostics: A Single-center Experience and Lessons Learnt

**DOI:** 10.5041/RMMJ.10341

**Published:** 2018-07-30

**Authors:** Karin Weiss, Alina Kurolap, Tamar Paperna, Adi Mory, Maya Steinberg, Tova Hershkovitz, Nina Ekhilevitch, Hagit N. Baris

**Affiliations:** 1 The Genetics Institute, Rambam Health Care Center, Haifa, Israel; 2 The Ruth and Bruce Rappaport Faculty of Medicine, Technion–Israel Institute of Technology, Haifa, Israel

**Keywords:** Consanguinity, personalized medicine, rare genetic disease, whole-exome sequencing

## Abstract

**Objective:**

The growing availability of next-generation sequencing technologies has revolutionized medical genetics, facilitating discovery of causative genes in numerous Mendelian disorders. Nevertheless, there are still many undiagnosed cases. We report the experience of the Genetics Institute at Rambam Health Care Campus in rare disease diagnostics using whole-exome sequencing (WES).

**Methods:**

Phenotypic characterization of patients was done in close collaboration with referring physicians. We utilized WES analysis for diagnosing families suspected for rare genetic disorders. Bioinformatic analysis was performed in-house using the Genoox analysis platform.

**Results:**

Between the years 2014 and 2017, we studied 34 families. Neurological manifestations were the most common reason for referral (38%), and 55% of families were consanguineous. A definite diagnosis was reached in 21 cases (62%). Four cases (19%) were diagnosed with variants in novel genes. In addition, six families (18%) had strong candidate novel gene discoveries still under investigation. Therefore, the true diagnosis rate is probably even higher. Some of the diagnoses had a significant impact such as alerting the patient management and providing a tailored treatment.

**Conclusions:**

An accurate molecular diagnosis can set the stage for improved patient care and provides an opportunity to study disease mechanisms, which may lead to development of tailored treatments. Data from our genetic research program demonstrate high diagnostic and novel disease-associated or causative gene discovery rates. This is likely related to the unique genetic architecture of the population in Northern Israel as well as to our strategy for case selection and the close collaboration between analysts, geneticists, and clinicians, all working in the same hospital.

## INTRODUCTION

In the past decade, the growing availability of next-generation sequencing technologies has revolutionized the field of medical genetics. The improvement in sequencing and analytic abilities led to a shift from hypothesis-driven investigations into hypothesis-free approaches in rare disease diagnostics. This has resulted in a significant improvement in the diagnosis rate of rare diseases.^1^ It also led to the identification of a wider phenotypic spectrum in known diseases, discovery of novel phenotypes associated with known genes, discovery of new rare diseases associated with novel genes not previously investigated, and blended phenotypes due to more than one diagnosis.^2^ There are numerous advantages to the identification of the genetic etiology in rare diseases: the specific diagnosis can alter case management and the recommendations for treatment and follow-up; it provides an end to the diagnostic odyssey that is time- and money-consuming; it facilitates family planning and counseling; and it triggers research on the disorder.

One of the most cost-effective tools in current use is whole-exome sequencing (WES), which targets all exons of protein-coding genes in the genome. The diagnostic yield of WES ranges between 25% and 50% depending on the clinical presentation and the number of previous investigations.^3^–^6^ Nevertheless, at least 50% of the cases with suspected Mendelian disorders remain undiagnosed, and recently there has been a decrease in the rate of novel gene and novel genotype–phenotype discoveries.^3^ The main bottlenecks to diagnostic success are related to the interpretation of the clinical and sequencing data, and the ability to perform functional investigations of novel candidate genes.

Here, we present the experience of the Genetics Institute at Rambam Health Care Campus (RHCC; Haifa, Israel) in rare disease diagnostics, and we describe the results of our research-based exome-sequencing program.

## METHODS

**Figure 1:**
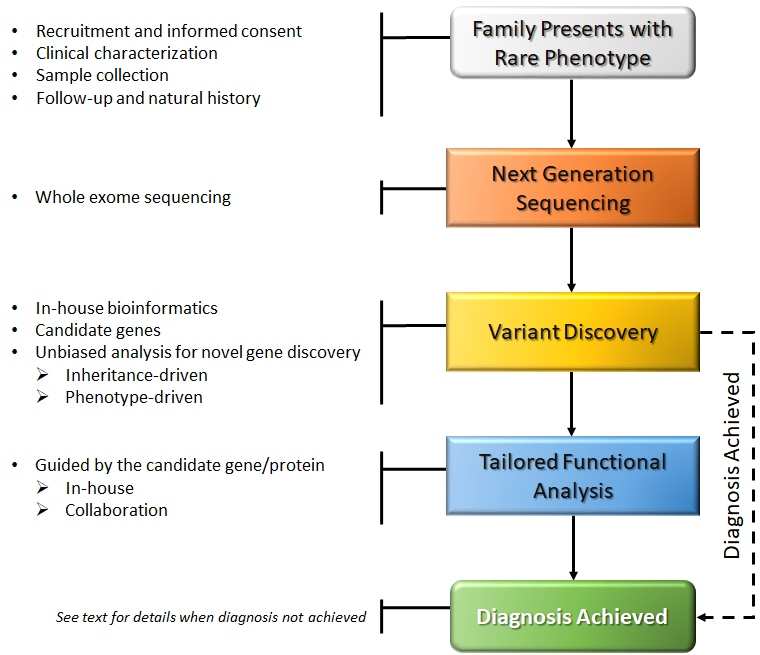
Pipeline of the Genetics Institute Rare Disease Research Program

In 2014 we established a research pipeline for rare disease diagnostics that serves the entire hospital and provides an opportunity to investigate unusual and complex medical cases from different disciplines, including the pediatric and adult populations. The pipeline strategy is summarized in Figure 1.

### Ethical Standards

A core protocol study for deciphering the genetic basis of rare monogenic disorders has been approved by the institutional Helsinki Committee. All participants received a full explanation regarding the study aims and procedures and signed a written informed consent form as customary.

### Patient Recruitment and Clinical Evaluation

Cases were selected based on a high clinical suspicion of a Mendelian disorder based on disease presentation and family history. We did not concentrate on a specific clinical presentation but rather included a variety of cases in collaboration with different subspecialists, such as gastroenterologists, endocrinologists, immunologists, oncologists, cardiologists, neurologists, metabolic specialists, and others.

Healthy and affected study participants were evaluated by medical geneticists and their referring physicians, including in-depth phenotyping and a detailed intake of family history and pedigree. Clinical data from medical records were used for further refinement of the studied phenotype, as well as for patient follow-up.

During the data analysis and interpretation stages, we were able to go back and re-evaluate patients, as well as consult with referring specialists on the results.

### Sample Collection and Genetic Analysis

We collected DNA samples from available affected and healthy family members and sent them for WES to one of several external laboratories: Gene by Gene (Houston, TX, USA), Macrogen (Seoul, South Korea), and Regeneron Genetics Center (Tarrytown, NY, USA) as part of an ongoing collaboration. The number of sequenced participants was determined individually for each family, depending on assumed inheritance pattern, family structure, and availability of samples.

Bioinformatic data analysis was performed in-house using the Genoox data analysis platform pipeline (Genoox Ltd, Tel Aviv, Israel). Data filtering was initially guided by the assumed inheritance pattern of the studied phenotype to include variants that are rare (minor allele frequency <0.01 in public and in-house variant databases), protein-altering (missense, nonsense, frameshift, splice-site), and segregated as expected among the sequenced family members. Candidate variants were prioritized based on gene function and relevance to the studied phenotype and were validated by Sanger sequencing.

## RESULTS

**Figure 2:**
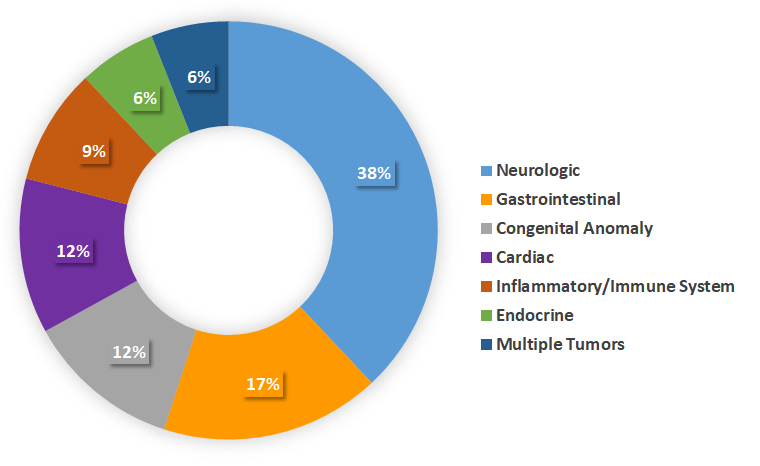
Distribution of Cases Referred to the Rare Disease Research Program by Medical Field

Between the years 2014 and 2017 we enrolled 34 families in our rare-disease research program. Results are summarized in Table 1; 55% of the families were consanguineous, and 44% had more than one affected individual. A neurologic phenotype was present in 38% of the cases we investigated. Other reasons for referral were infantile colitis, protein-losing enteropathy, cardiomyopathy, congenital anomalies, multiple tumors, and endocrine and immunologic abnormalities (Figure 2).

**Table 1:** Summary of Results

Analyses Result	Families *n* (%)	Consanguinity *n* (%)	Multiple Affected *n* (%)	Homozygous Recessive *n* (%)	Compound Recessive *n* (%)	Autosomal Dominant *n* (%)	Neurologic Phenotype *n* (%)
Definite Diagnosis	21 (62%)	14 (66%)	12 (57%)	14 (66%)	1 (4%)	6 (28%)	8 (38%)
Candidate	6 (18%)	2 (33%)	2 (33%)	2 (33%)	2 (33%)	2 (33%)	3 (50%)
No Diagnosis	7 (20%)	3 (42%)	1 (14%)	NA	NA	NA	2 (28%)
Total	34 (100%)	19 (55%)	15 (44%)	16 (47%)	3 (8%)	8 (23%)	13 (38%)

NA, not applicable.

**Figure 3:**
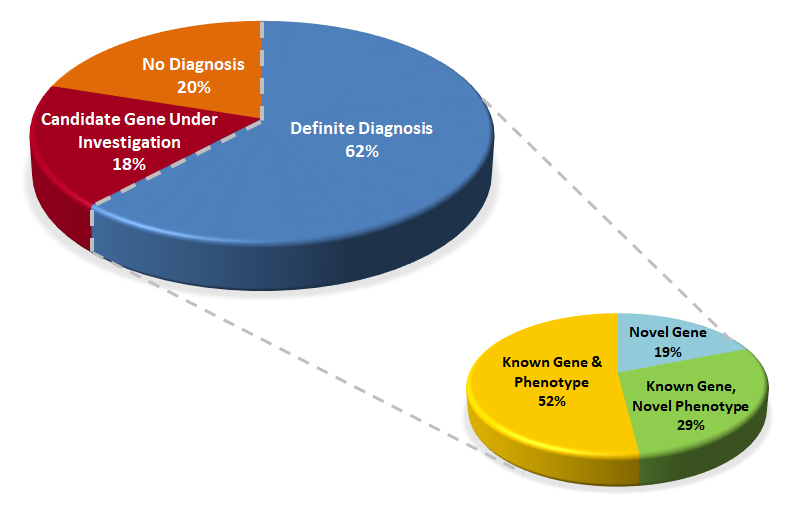
Diagnostic Rates in 34 Families Studied at the Genetics Institute from 2014 to 2017 with Novel Gene Discovery Rate (lower chart)

A definite diagnosis was obtained for 21 families (62%) (Table 1, Figure 3); 17 cases (50%) were diagnosed with variants in known disease-causing genes, of which six demonstrated a novel phenotype, and two families had a double diagnosis.

Moreover, four cases (19%) were diagnosed with variants in novel genes, prompting the establishment of research projects that proved their pathogenicity. In addition to the cases with a definite diagnosis, six families (18%) had strong candidate gene discoveries, still under investigation. Therefore, the true diagnosis rate is predicted to be even higher, up to 80%, with a potential for identification of 10 novel genes (29%) overall. When comparing the group with a definite diagnosis to the entire cohort, there was a trend toward a higher rate of consanguinity, multiple affected individuals in one family, and homozygous recessive mode of inheritance. The presence of a neurologic phenotype was not more frequent in the definite diagnosis group. Of note, autosomal dominant and compound heterozygous recessive inheritance was observed in 2 out 14 of consanguineous families (14%), and homozygous recessive inheritance was seen in 2 out of 7 families (14%) with no known consanguinity possibly due to a founder mutation.

## DISCUSSION

Rambam Health Care Campus is the only tertiary medical center serving Northern Israel. It serves a uniquely diverse population of different ethnicities, including Jews of different backgrounds, Muslim Arabs, Christian Arabs, Druze, Circassians, and others. For some of these ethnic groups, marriage within the family (consanguinity), village or ethnic group (endogamy) is a common practice. These result in identity-by-descent and an increased frequency of homozygous recessive disorders. It is estimated that, out of over 7,000 rare genetic diseases, approximately half have been genetically diagnosed, leaving a bulk of unrecognized disease-causing genes.^3^ The unique population that we encounter facilitates novel gene discovery that aids in narrowing this gap, as exemplified by the high rate of novel gene discovery in this cohort.

It has been previously reported that consanguinity may increase the diagnostic yield of exomes.^7^,^8^ This could be due to a more straightforward analysis or a higher propensity of genetic etiologies in these populations. However, caution should be exercised as we identified an alternative mode of inheritance (autosomal dominant or compound het) in two consanguineous families. Powis et al. found an even higher incidence of non-homozygous inheritance in consanguineous pedigrees.^9^ Conversely, homozygous recessive inheritance was seen in two cases, where the parents shared the same ethnic origin but there was no known consanguinity. Another phenomenon that should be emphasized is the possibility of a double diagnosis, which was noted in two families in our cohort. The fusion of two phenotypes might direct the physician to an erroneous diagnosis and work-up. In a previous publication we reported that founder mutations and consanguinity are two factors that should raise the index of suspicion for a double diagnosis.^10^ These possible diagnostic errors emphasize the importance of a non-biased analysis provided by WES.

Traditionally many of the large sequencing projects concentrate on neurologic and neurodevelopmental phenotypes.^11^,^12^ Here we show a similar diagnostic yield with non-neurologic phenotypes, supporting the importance of genomics in different fields in medicine.

Reaching a molecular diagnosis provides several advantages, among which is the knowledge gained on novel pathological pathways. These can, at times, direct the surveillance and care, as well as enable the offering of prenatal diagnosis to the couple, allowing for primary prevention. Furthermore, deciphering the cause of a rare disease can shed light on the pathogenesis of the disorder, and can even provide new insights yielding novel treatment options. This was well established in lipid disorders and the *PCSK9* gene, with the discoveries that gain-of-function variants cause hypercholesterolemia, but rare homozygous loss-of-function mutations lead to lower cholesterol levels without any apparent health consequences. These findings stimulated the development of novel anti-PCSK9 drugs for hypercholesterolemia.^13^

In this cohort we identified 10 potential novel genes. There are several possibilities for determining their role in human disease and the effect of the specific mutations identified. Animal models such as the fruit fly, zebrafish, and mouse can be utilized to recapitulate the phenotype in humans. This can be achieved with knock-out studies or gene editing for a specific missense variant. In addition, cell studies can also determine the effect of genetic variants on protein function, protein expression, splicing, and others.

To illustrate the benefits of our approach, we present three families serving as a model for our mode of research:

One of the immediate goals of our initiative is to provide the families with a molecular diagnosis, allowing them to pursue prenatal testing or preimplantation genetic diagnosis (PGD), all done in our center. An example for this was the diagnosis of GLYT1-encephalopathy, caused by bi-allelic mutations in *SLC6A9*, in a single child born to healthy double-first cousins. Her phenotype was similar to glycine encephalopathy, but she had normal levels of serum glycine and only mildly elevated cerebrospinal fluid glycine.^14^ Interestingly, she had a homozygous loss-of-function variant in a glycine transporter gene not previously known to be associated with disease. Following this diagnosis, the parents underwent *in vitro* fertilization and PGD, leading to the birth of a healthy boy. Moreover, the diagnosis in the first family led to a retrospective diagnosis for a family that was cared for in RHCC during the 1990s. In this family, three babies died at ages 2 days to 7 months, displaying a very similar phenotype to the proband in the first family. Sanger sequencing of the gene in DNA available from one affected child revealed a different homozygous truncating variant in the same gene. This allowed closure for the family and accurate counseling regarding the recurrence risk for relatives.

Another major aim that may be approached by genetic diagnosis is to implement personalized medicine in the clinic. One unique example allowed us not only to genetically diagnose a family but also to tailor treatment based on the molecular findings by repurposing a commercial drug. Using WES in an extended family with multiple individuals affected with early-onset protein-losing enteropathy and major thrombotic events, we were able to isolate a single pathogenic variant in the complement regulator CD55, segregating with the disease.^15^ This finding was supported by another group describing several families with various mutations in *CD55*.^16^ Over the years, the patients were treated only by dietary restrictions, and were often hospitalized due to abdominal complications; two family members, including a 4-year-old girl, died due to disease complications. The diagnosis of CD55-deficiency, together with demonstration of abnormal complement activation on patient leukocytes, led to a successful compassionate treatment of three patients with the terminal complement inhibitor eculizumab.^15^ If not for the genetic diagnosis that allowed for the drug repurposing, one of the affected children, who was in a critical condition, would have died.

The ability to diagnose the patients within a treating hospital also allows for better care and surveillance. Two siblings, a boy and a girl, born to first-degree cousins of Druze descent presented with multiple congenital anomalies, including trachea-esophageal fistula, cardiac defects, renal and spinal anomalies, suggestive of VACTERL association (vertebral anomalies, anorectal malformations, cardiovascular anomalies, tracheoesophageal fistula, esophageal atresia, renal and/or radial anomalies, limb defects). Exome sequencing identified a homozygous pathogenic variant in *FANCA*, causing Fanconi anemia. Their genetic diagnosis prompted referral to the hematology clinic, with immediate diagnosis of pancytopenia in one sibling; she is now planned to undergo bone marrow transplantation. In this case, the rapid genetic diagnosis of this serious condition shortened the time for appropriate care, thus preventing unnecessary evaluations.

While this report is of limited extent, we believe it reflects and represents a true high diagnostic yield, compared to the previously reported rates in WES. The high yield can be attributed to several factors. First, the selection of patients with unusual phenotypes and a high degree of suspicion for a Mendelian disorder; second, the inclusion of families with high rates of identity by descent, and informative families with more than one affected individual, which facilitated data analysis; lastly, the phenotyping and data analysis were done in the same center, in close collaboration with physicians and other medical professionals within the hospital. This enabled us to perform more accurate phenotyping contributing to improved diagnostics.

In summary, our research approach consisting of a close collaboration between the clinics and the genetics laboratory has yielded a relatively high genetic diagnostic rate. The genetic diagnoses have benefited patients and their families by improving medical care and enabling prevention through prenatal and preimplantation diagnoses.

## Abbreviations

DNA: deoxyribonucleic acid
NA: not applicable
PGD: preimplantation genetic diagnosis
RHCC: Rambam Health Care Campus
VACTERL: vertebral anomalies, anorectal malformations, cardiovascular anomalies, tracheoesophageal fistula, esophageal atresia, renal and/or radial anomalies, limb defects
WES: whole-exome sequencing.

## References

[1] Yang Y, Muzny DM, Xia F (2014). Molecular findings among patients referred for clinical whole-exome sequencing. JAMA.

[2] Sobreira NL, Valle D (2016). Lessons learned from the search for genes responsible for rare Mendelian disorders. Mol Genet Genomic Med.

[3] Boycott KM, Rath A, Chong JX (2017). International cooperation to enable the diagnosis of all rare genetic diseases. Am J Hum Genet.

[4] Córdoba M, Rodriguez-Quiroga SA, Vega PA (2018). Whole exome sequencing in neurogenetic odysseys: an effective, cost- and time-saving diagnostic approach. PLoS One.

[5] Retterer K, Juusola J, Cho MT (2016). Clinical application of whole-exome sequencing across clinical indications. Genet Med.

[6] Sawyer SL, Hartley T, Dyment DA (2016). Utility of whole-exome sequencing for those near the end of the diagnostic odyssey: time to address gaps in care. Clin Genet.

[7] Alfares A, Alfadhel M, Wani T (2017). A multicenter clinical exome study in unselected cohorts from a consanguineous population of Saudi Arabia demonstrated a high diagnostic yield. Mol Genet Metab.

[8] Fattahi Z, Kalhor Z, Fadaee M (2017). Improved diagnostic yield of neuromuscular disorders applying clinical exome sequencing in patients arising from a consanguineous population. Clin Genet.

[9] Powis Z, Farwell KD, Alamillo CL, Tang S (2016). Diagnostic exome sequencing for patients with a family history of consanguinity: over 38% of positive results are not autosomal recessive pattern. J Hum Genet.

[10] Kurolap A, Orenstein N, Kedar I (2016). Is one diagnosis the whole story? Patients with double diagnoses. Am J Med Genet A.

[11] Wright CF, Fitzgerald TW, Jones WD (2015). Genetic diagnosis of developmental disorders in the DDD study: a scalable analysis of genome-wide research data. Lancet.

[12] Allen AS, Berkovic SF, Cossette P, Epi4K Consortium; Epilepsy Phenome/Genome Project (2013). De novo mutations in epileptic encephalopathies. Nature.

[13] Cohen JC, Hobbs HH (2013). Simple genetics for a complex disease. Science.

[14] Kurolap A, Armbruster A, Hershkovitz T (2016). Loss of Glycine transporter 1 causes a subtype of glycine encephalopathy with arthrogryposis and mildly elevated cerebrospinal fluid glycine. Am J Hum Genet.

[15] Kurolap A, Eshach-Adiv O, Hershkovitz T (2017). Loss of CD55 in eculizumab-responsive protein-losing enteropathy. N Engl J Med.

[16] Ozen A, Comrie WA, Ardy RC (2017). CD55 deficiency, early-onset protein-losing enteropathy, and thrombosis. N Engl J Med.

